# Community patterns of the small riverine benthos within and between two contrasting glacier catchments

**DOI:** 10.1002/ece3.679

**Published:** 2013-07-22

**Authors:** Ursula Eisendle-Flöckner, Christian D Jersabek, Martin Kirchmair, Kerstin Hashold, Walter Traunspurger

**Affiliations:** 1 Division of Animal Structure and Function, Department of Cell Biology, University of SalzburgHellbrunnerstraße 34, 5020, Salzburg, Austria; 2 Institute of Microbiology, University of InnsbruckTechnikerstraße 25, 6020, Innsbruck, Austria; 3 Department of Animal Ecology, University of BielefeldMorgenbreede 45, 33615, Bielefeld, Germany

**Keywords:** Algae, bacteria, copepods, fungi, glacier retreat, glacier river, local patterns, meiofauna, nanoflagellates, nematodes, regional diversity, rotifers

## Abstract

Ongoing glacial retreat is expected to lead to numerous changes in glacier-fed rivers. This study documents the development of community composition of the hitherto widely neglected micro- and meiobenthos (MMB: bacteria, fungi, algae, protists, and meiofauna) in glacier rivers in response to the distinct habitat conditions driven by different stages of (de)glacierization. Our model is based on the glacier catchments of the Möll River (MC) and Kleinelendbach stream (KC), in the Austrian Alps, with 60% and 25% glacierization and glacier retreats of 403 and 26 m, respectively, since 1998. Analyses of overall catchment diversity and resemblance patterns showed that neither intense glacierization nor rapid deglacierization were predominant MMB determinants. This was ascribed to the specific environmental conditions at the MC, where the rapidly retreating Pasterze glacier has formed a harsh unstable proglacial, but also a benign floodplain area, with the former suppressing and the latter supporting the structural development of the MMB. Comparisons of similarly aged riverine habitats of the MC proglacial and the KC main channel further evidenced developmental suppression of the MMB (64 taxa) by the rapidly retreating MC glacier, unlike the moderate glacial retreat in the KC (130 taxa). Habitat conditions interacting with melt periods explained the differences in MMB resemblance patterns, which themselves differentially reflected the spatiotemporal habitat settings imposed by the different glacier activities. The varying glacial influences were represented by a glaciality index (GIm) based on water temperature, electrical conductivity, and stream bed stability. The taxonomic richness of nematodes, rotifers, algae, and diatoms was distinctly related to this index, as were most MMB abundances. However, the strongest relationships to the GIm were those of nematode abundances and maturity. Our observations highlight the intense response of the MMB to ongoing glacier retreat and the utility of a simple index to reveal such patterns.

## Introduction

Riverine habitats of glacier catchments are among the many highly vulnerable habitats that are threatened by present-day glacier retreat patterns and global climate change (Woodward et al. 2010; Vanham 2012). This threat becomes even more alarming given the far reaching consequences expected for riverine biota, whose complexity and complex relationships still constitute major contemporary “black boxes” in ecological global change research. In general, river research has suffered from an imbalance in that it has largely focused on just a few organismal groups, mostly belonging to the macrofauna (Milner et al. 2001, 2009; Brown et al. 2009, 2010; Finn et al. 2010; Parker and Huryn 2011; Jacobsen and Dangles 2012), rather than on complex community patterns (Stendera et al. 2012). However, baseline data for all glacier river inhabitants are required, not only to ensure reliable evaluations of the potential response of riverine biota to glacial and climate changes but also to enable progressive and therein better targeted research into the resulting altered relationships between biota components. This in turn will allow estimations of ecosystem performances, now and in the future (Lecerf and Richardson 2010).

Glaciers act as a major determinant of their catchment's riverine habitats, where a large degree of glacierization (Füreder 2007) as well as ablation activity accounts for the harshness within these environments (McGregor et al. 1995) While glaciers and their climate responses have become better understood over the course of decades of research, which predicts ongoing glacier retreat (WGMS 2008; Dessler and Parsons 2010), uncertainties remain due to the complex reciprocity between climate, glaciers, and their surroundings (Pelto and Brown 2012). The uniqueness of each glacier is further exemplified by the variety of developmental stages, hydrological regimes, and habitat heterogeneity (templates and patchiness) within alpine riverine networks following glacial retreat in response to ongoing climate change. In fact, major changes are expected for catchments with a high degree of glacierization and for glaciers with significant ablation zones (Hook 2005; Dyurgerov et al. 2009; Farinotti et al. 2012). Thus, after an effective increase in glacier influence following major loss of ice masses, distinct shifts in the water supply are expected (Milner et al. 2009). This will cause temporary (particularly in summer) but also permanent desiccation within riverine sectors due to the resulting absence of direct (melt water effluent) and indirect (replenishing groundwater storage) glacier influences (Tockner and Malard 2003).

In glacier-fed rivers, the relatively long history of research targeting the macrozoobenthos has produced important insights, such that its community patterns (e.g., species composition and abundances, and ecological traits) have been related to riverine habitat conditions subject to the varying influences of the glaciers that supply them (Ilg and Castella 2006; Füreder 2007; Brown et al. 2010; Finn et al. 2010; Muhlfeld et al. 2011; Jacobsen and Dangles 2012). Thus, changes in climate and glacierization are expected to result in corresponding changes, including species extinction, in the macrozoobenthos of distinctly glacier-driven habitats. For other organismal groups comprising the benthic biota of these habitats, our limited knowledge has hindered an understanding of their relationships with abiotic and biotic characters and of the expected alterations thereof in response to ongoing climate change (Milner et al. 2009). This deficit derives from the widely substrate-biased observations of algae (epilithic; Rott et al. 2006) and fungi (litter traps; Gessner and Robinson 2003) and the paucity of studies of other groups such as bacteria (Battin et al. 2001, 2004) and the meiofauna (Eisendle 2008).

Among the meiobenthos, nematodes act as a relatively diverse colonizer at the glacier source and contribute, both in terms of abundance and diversity, to the distinct habitat templates further downstream (e.g., proglacial, glacio-rhithral) (Eisendle 2008). These findings suggest the important participation of nematodes as mediators in nutrient cycling processes in glacier-fed rivers, as similar to that presumed for algae as primary producers and for bacteria and fungi as decomposers. This role as essential nutrient mediators is a function of both the broad resource usage of nematodes and their many predators (Traunspurger 2002; Beier et al. 2004; Muschiol et al. 2008; Spieth et al. 2011). However, it can also be assumed that nematodes, and members of the meiobenthos in general, are contributing factors to the as yet poorly understood resources of macrozoobenthic consumers in glacier rivers (Füreder et al. 2003). Consequently, the involvement of the meiobenthos in food-web relationships and ecosystem performance must be considered in any discussion of the changes predicted for glacier-fed rivers and their biota.

In an unprecedented approach, this study investigated the hitherto widely neglected micro- and meiobenthos (MMB: bacteria, fungi, algae, protists, nematodes, rotifers, copepods, tardigrades) in the Möll River catchment (MC) and the Kleinelendbach stream catchment (KC), both located in the Austrian Alps. These two differently glaciated catchments are representatives of past and ongoing glacier retreat patterns. Thus, in the MC, the large ablation zone of the dominant Pasterze glacier has been rapidly retreating whereas the slow retreat of the KC glaciers over the last few decades reflects previous major losses of their ablation zone. Overall, the MC environment is considered to be harsher than that of the KC mainly due to the extent of glacierization and the rapidly retreating ice masses. The two catchments have established riverine reaches that greatly differ in terms of harshness, a consequence of the spatiotemporally varying influences of their respective glaciers. Accordingly, we hypothesized: (1) significant differences in the MMB between the catchments, their reaches, and the relevant seasons (before, during, and after the glacier melt), and (2) that the changing habitat conditions produced by the varying glacier influences would be accompanied by significant alterations in the MMB.

## Material and Methods

### Study sites

**Figure 1:**
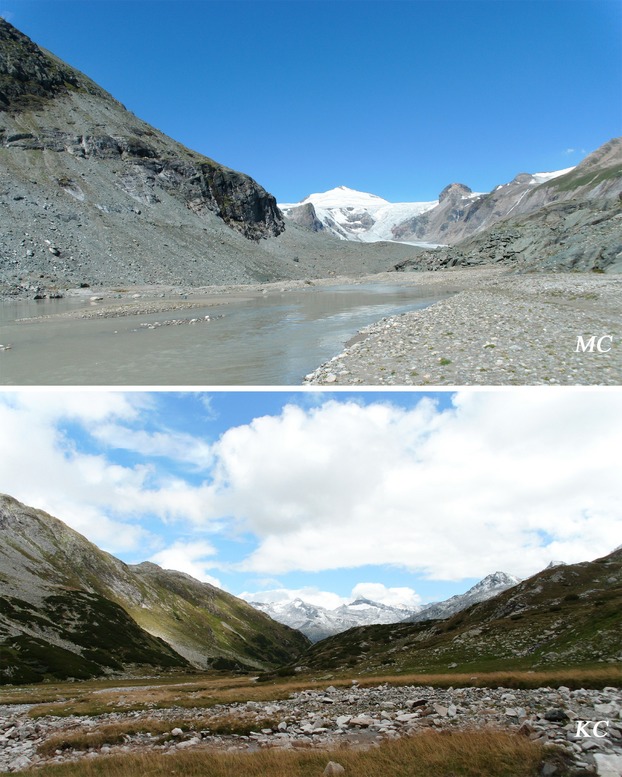
Photographs of the Möll River (MC) and Kleinelendbach stream (KC) catchments.

**Figure 2:**
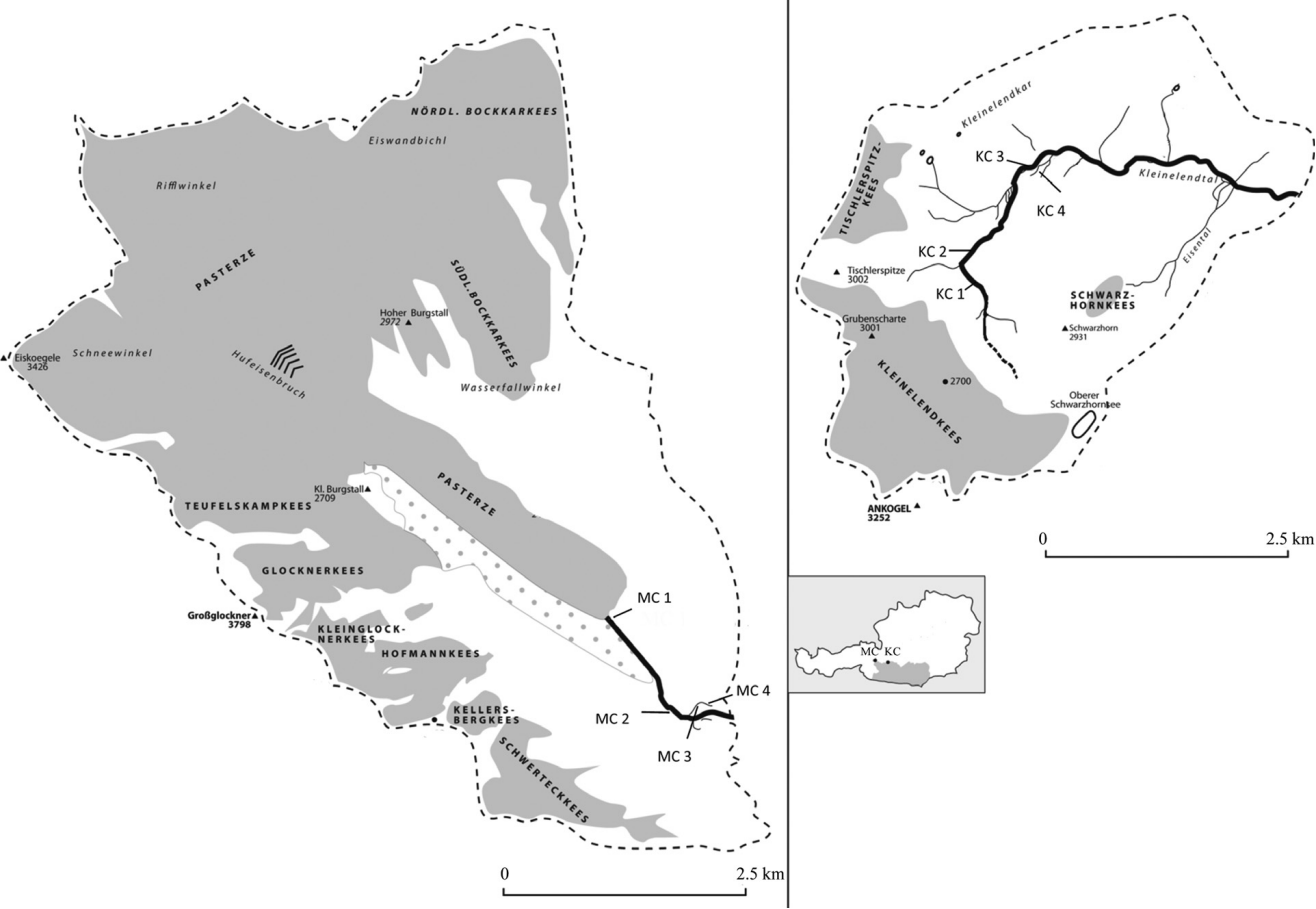
Studied river reaches (MC1–MC4, KC1–KC4) within the two catchments (MC, Möll River catchment; KC, Kleinelendbach stream catchment) in the Eastern Alps (Austria).

The MC and the KC (Nationalpark Hohe Tauern, Eastern Alps, Austria; Figs. 1, 2) were investigated at the beginning (GMI), middle (GMII), and end (GMIII) of the glacier melting period in 2010. The two catchments mainly differ in their areas, degree of glacierization, number of glaciers, and glacial retreat patterns, both during the past several decades and throughout the study period (Table 1). These differences imply different stages of succession and the distinct habitat conditions of the investigated river reaches (Table 2), which cover the youngest as well as the oldest potentially accessible sectors with respect to present-day glacial retreat and height during the Little Ice Age (1850 AD). At each catchment, a water gauge was installed to measure water temperature (°C), pH, electrical conductivity (μS cm^−1^), and turbidity (NTU) (MS5, Otthydromet, Kempten, Germany). Discharge data for the MC were obtained from the Hydropower Agency and water level data at the KC from an additional gauge (Data logger, Orpheus Mini, Otthydromet). The vegetation of the two catchments consists mainly of alpine meadows.

**Table 1:** Characteristics of the Möll River (MC) and Kleinelendbach stream (KC) catchments

	MC	KC
Geographic coordinates (N/E)	47°04′/12°44′	47°04′/13°16′
Catchment area (km²)	36	12
Number of glaciers	9	2
Glacier area (km²)	22	3
Percentage glacierization	61	25
Main glacier	Pasterze glacier	Kleinelendkees glacier
Retreat 2010 (m)	40	2.7
Retreat since 1998 (m)	403 (debris-free tongue)	26

**Table 2:** Abiotic characters of the river reaches: altitude (ALT, m asl.), distance to uppermost glacier margin (DG, km), reach age (RA, years since deglacierization; data according to Lang 1993 for KC and according to Lieb and Slupetzky [2004] for MC), and stream width (SW, m). Point measurement ranges during the sample period 2010 were determined for water temperature (T, °C), electrical conductivity (EC, μS cm^−1^), pH, oxygen (O_2_, %), and the bottom index (BI; according to Pfankuch [1975]). Ranges and averages of gauge measurements (in parentheses) at KC and MC from July to August 2010: T, EC, and pH; O_2_ is replaced by turbidity (*NTU) for the gauge measurements. WD, WV = mean water depth (m) and velocity (m sec^−1^); *n* = 22 for MC1 and 45 for other reaches. GI_m_, mean values of the modified glaciality index for each reach

	ALT	DG	RA	SW	T	pH	EC	BI	O_2_	WD	WV	GI_m_
KC1	2241	0.5	35	3–6	7.9–9.3	7.9–8.3	6–70	44	93–105	0.16	0.09	−0.51
KC2	2190	1.9	70	3–10	4.3–9.4	7.4–8	10–65	33	99–102	0.16	0.08	−0.11
KC3	2112	1.9	∼160	3–10	5–7.5	8.18–8.23	8–38	33	95–104	0.23	0.16	−0.27
KC4	2112	1.2	∼160	0.5–1	8.3–11.8	7.3–7.6	74–120	21	98–104	0.09	0.01	1.24
MC1	2100	0	0	1–3	0.3–0.5	8.1–8.3	5–28	58	97–98	0.10	0.23	−1.54
MC2	2070	0.9	30	3–7	0.6–1.4	7.9–8.6	28–90	51	97–98	0.47	0.07	−0.88
MC3	2070	1.2	48	0.5–1	8.8–14.5	8.1–8.8	115–267	25	98–99	0.19	0.002	1.04
MC4	2070	1.3	54	0.5–1	6–15.4	7.9–8.05	64–270	25	97–101	0.13	0.0	1.02
KC					0.3–12.5 (6.3)	6.9–7.8 (7.4)	9–34 (23)		*NTU 1–**369 (4)**			
MC					0.03–6.1 (4.4)	6.2–11.0 (8.9)	0–129 (34)		*NTU 1–**3000 (515)**			

### Field work

Habitat stability was evaluated by the bottom component of the Pfankuch index (BI [bottom stability], Pfankuch 1975) for each date and reach. Point measurements (water temperature [°C], pH, electrical conductivity [μS cm^−1^], O_2_ [%]; measuring equipment: HI-98107pHep, Hanna Instruments and Multi 3420, WTW, Weilheim, Germany) and PVC corer samples (benthic microbiota [*n* = 4; sampling area: 4 cm^2^], fungi [*n* = 5; 12.56 cm^2^], protists and algae [*n* = 2; 12.56 cm^2^], and invertebrates [*n* = 5; 12.56 cm^2^] of the upper river bed sediments [0–10 cm]) were obtained for each date and reach, except MC3 and MC4. At the latter, because of the homogenously silted habitat, a smaller corer (4 cm^2^) was used for sampling each organismal group. Additionally, 6–10 stones were brushed for diatom samples (Rott et al. 2006), except at MC3 and MC4, where about 1 cm^2^ of the uppermost epipelic substrate was removed with a spoon. After sampling, water depth (WD, m) and water velocity (WV, m^−sec^; GMH 3350, Fm. Greisinger, Regenstauf, Germany) were determined for the corer samples. Organismal groups were processed according to the methods listed in Table S1.

### Modified glaciality index (GI_m_)

As in previous studies of glacial river macroinvertebrates by other authors (Ilg and Castella 2006; Brown et al. 2010), we calculated a glaciality index (GI), albeit in a modified version (GI_m_). The GI_m_ integrates three habitat characteristics that change with changing glacier influence, namely, water temperature (T), electrical conductivity (EC), and BI. The reciprocal of the BI (BI_rez_) was yet used in the GI_m_ to ensure that all parameters therein decrease with increasing glacier influence. The three variables were standardized before running a noncentric principal components analysis (NPCA), whose first-axis ordination scores yielded the GI_m_ values for each sampling occasion (each reach at each date).

### Community characters

Microbiota morphotypes were classified into cocci, rods, unbranched filamentous bacteria (UFB) (size classes of ≤10 and >10–20 μm), cyanobacterial colonies and threads, and heterotrophic and autotrophic flagellates (size classes of ≤3, >3–6, >6–10, >10 μm). Fungal CFU (colony-forming units) counts were measured as the total for each replicate and for the dominant species on each sampling occasion. Fungal species numbers were determined in total for each reach. Abundances were defined at the genus level for protists and for algae in the Bouinse samples. Nematode (five replicates) and rotifer (three to five replicates) species abundances were determined whenever possible; invertebrate abundances were determined based on coarse taxonomic resolution (five replicates).

Nematode maturity was calculated as MI = ∑ *v*_i_ × *p*_i_ (Bongers 1990), where *v*_i_ is the *c*-*p* value, and *p*_i_ the frequency of taxon i. The Shannon diversity of nematodes and rotifers was calculated as H′ -∑_i_ *p*_i_ log (*p*_i_), where *p* is the frequency of taxa_i_. Diatom diversity is given in species numbers.

### Data analysis

We first tested whether catchment, reach, and season or a combination thereof accounted for significant differences in the community structure of each group of organisms (microbiota morphotype, fungal CFU counts, algae/protists complex, invertebrates, and nematode and rotifer species). As the respective data did not meet parametric requirements (normal distribution, variance homogeneity), a nonparametric three-way analysis of variance (PERMANOVA; factors: catchment, reach, season) was applied based on the Bray Curtis resemblance matrix of the square-root-transformed data of each organismal group. The null hypothesis (no significant influence of any factor or of any interaction between them) was rejected at *P* ≤ 0.01 (9999 permutations). To avoid negative estimates of variation, we pooled the factor “catchment” for certain groups (microbiota, invertebrate coarse level) within the PERMANOVA routine (Anderson 2001). The PERMANOVA settings were as follows: partial sums of squares, reduced model for residuals permutation (except nondiatom algae and protists, and rotifers: unrestricted permutation due to replicate number), fixed effects sum to zero for mixed terms and a nested design with catchment and date as fixed factors, and reach factor nested in catchment.

The GI_m_ fulfilled parametric requirements (normal distribution; homogeneity of variances) and a one-way ANOVA (*P* < 0.01) was used to test for significant differences between the reaches, with a post hoc test (Tukey-HSD-Test; *P* < 0.05) used to identify distinct groupings therein. A potential relationship between habitat-specific glacial influence (GI_m_) and community parameters (abundances, taxa richness, biotic indices) was evaluated by regression analysis. This step was preceded by using correlation patterns to determine the linearity between raw and transformed organism data versus the GI_m_. The parametric requirements of the residuals (normality, homoscedasticity) were first tested by graphical evaluation, followed by a Breusch–Pagan test to exactly evaluate deviations from homoscedasticity. These analyses confirmed the validity of using untransformed taxonomic richness (all groups except protists) and log10 (x + 1)-transformed abundance data, with the exception of the reciprocals of rotifer mean abundances in the regression analysis.

The evaluation of potential relations between MMB community structure and abiotic habitat conditions was complemented by correlation analyses using Spearman's rho to test for: (1) relationships between stream patchiness descriptors (WV, WD) and community characters (abundances, taxonomic richness, and biotic indices), and (2) relations between the resemblance matrices of the complex community structure of all organismal groups (except epilithic diatoms) and basic habitat factors (T°, EC, BI, mean WT, and WV) as determined by BIOENV analysis. Therein, the Bray–Curtis matrix of the log10 (x + 1)-transformed averages of all organisms groupings (except epilithic diatoms) of each sampling occasion was used together with the abiotic matrix (Euclidean distance) including hydrochemistry, mean WD and WV, and the BI after normalization (WV, WD, and conductivity were square-root-transformed prior to normalization). The BIOENV output yields a global significance level for Spearman's rho and the variables involved in its calculation.

Statistics were performed with SPSS (descriptive part, Spearman rank correlation, Chicago, IL), PRIMER 6.1.13 and PERMANOVA 1.0.3 (PRIMER LTD 2009, Plymoth, U.K.).

## Results

### Abiotic characters

**Figure 3:**
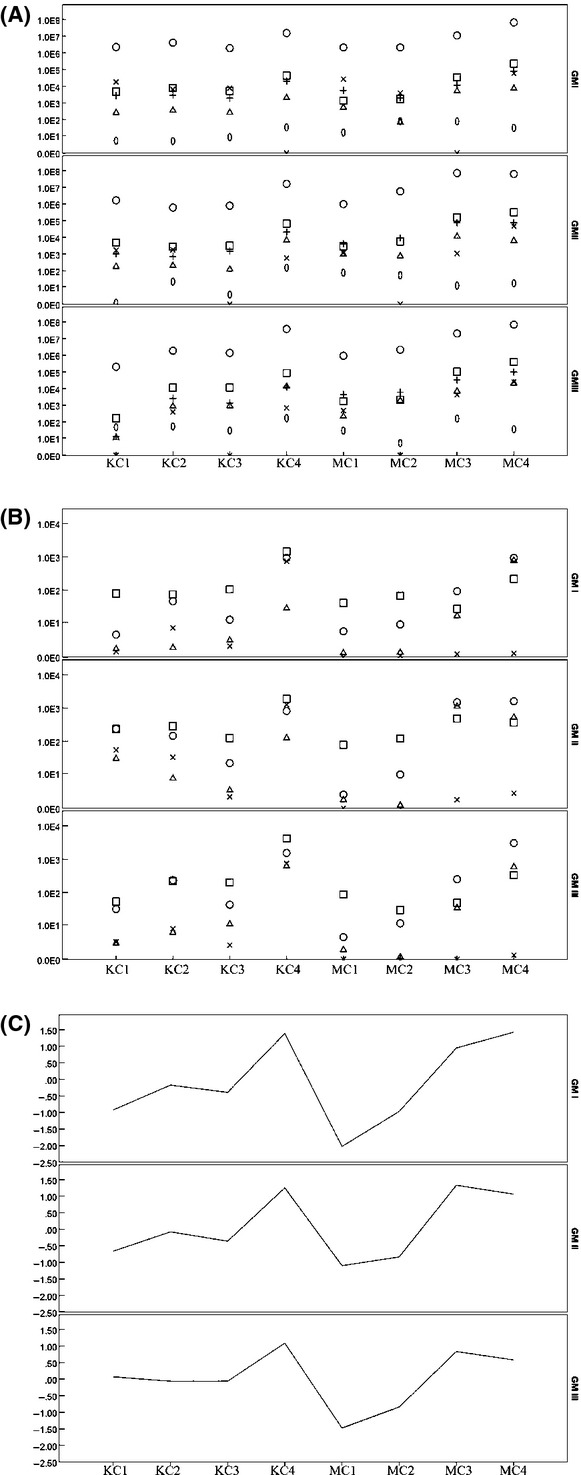
Spatiotemporal distribution of (A) counts of microbiota as (○, bacteria; □, unbranched threads; x, cyanobacteria; Δ, HNF; +, ANF – all Counts/cm^2^/mL; 0, fungal CFU); (B) abundances of dominant invertebrate groups (○, nematodes; □, rotifers; x, copepods; Δ, tardigrades; Ind/10 cm^2^/mL); and (C) the GI_m_.

Analyses of water temperature and turbidity indicated a minor influence of glacial ablation for the main channel of the KC compared to that of the MC (Table 2). Based on water temperature and BI, extremely harsh conditions were determined to prevail in the proglacial complex (MC1, MC2), harsh conditions along the KC main channel sites (KC1–KC3), more favorable (benign) conditions for the sandur complex (MC3 and MC4), and the least harsh conditions at KC4 (Table 2). There was no obvious longitudinal trend of habitat amelioration along the KC main channel. The site-specific harshness or absence thereof was also well reflected by the GI_m_ (Fig. 3C; Table 2). Representing the NPCA first-axis scores it explained 70% of the variability, with higher loadings for BI_rez_ (0.939), and water temperature (0.854) than for EC (0.686). The GI_m_ significantly differed between the investigated reaches (ANOVA: *F* = 30.8, *P* < 0.01_df = 7_), distinguishing, with only one overlap, three groups of river reaches (post hoc Tukey test; *P* < 0.05): the MC proglacial (MC1 and MC2), the main channel reaches (MC2, KC1–KC3), and the low-flow reaches (MC3, MC4, and KC4).

### Overall catchment and reach observations

In total, the KC harbored more species than the MC, which was characterized by less diverse assemblages for all groups but diatoms (Table 3). More than double the number of invertebrate and algal (other than diatoms) taxa were present in the KC than in the MC, while the number of protist genera nearly tripled from the MC to the KC. Species numbers were highest for diatoms, followed by nematodes and rotifers, and lowest for protists. The GI_m_ reach-specific grouping was better reflected by nematode maturity than by other diversity measures (Table 3), which were nonetheless lower in the main channel reaches of the MC than in those of the KC, where they did not consistently increase downstream. Most of the diatom (MC3) and nematode (MC4) species inhabited the sandur complex. The lowest total taxonomic richness was not observed at the youngest (MC1), but at the second youngest reach (MC2), which although nearly the same age as KC1 had half the number of taxa than KC1. The difference between the similarly aged KC3 and KC4 was also considerable but not as large.

**Table 3:** Taxon numbers summed for each reach and catchment as genus numbers for algae other than diatoms (AlgO; incl. cyanobacteria) and protists (PRO) and species numbers for other organisms (FUN, fungi; DIA, diatoms; ROT, rotifers; NEM, nematodes). The genera numbers of the latter are given in parentheses. MI, nematode maturity; INV, higher invertebrate taxa level; H′, Shannon diversity for the respective groups; SN, sums of the taxa for each reach and catchment

	FUN	DIA	AlgO	PRO	ROT	H′ ROT	NEM	H′ NEM	MI	INV	SN Tot
KC1	14 (9)	46 (24)	14	5	12 (6)	0.1	27 (19)	2.4	2.3	12	130
KC2	15 (9)	60 (29)	15	7	23 (9)	0.4	34 (20)	2.3	2.3	13	167
KC3	14 (7)	35 (24)	14	6	19 (9)	0.3	39 (25)	2.5	2.2	12	139
KC4	15 (9)	71 (33)	19	10	27 (12)	1.7	40 (21)	2.7	2.9	13	195
MC1	15 (10)	27 (18)	1	2	6 (3)	0.1	18 (12)	2.6	2.0	3	72
MC2	12 (7)	26 (14)	1	1	1 (1)	0	19 (13)	2.1	2.0	4	64
MC3	12 (8)	91 (36)	6	3	20 (9)	1.1	37 (21)	1.6	2.6	3	172
MC4	9 (6)	84 (36)	5	1	17 (8)	1.2	44 (23)	1.5	2.6	6	166
KC	29 (13)	108 (39)	20	11	39 (14)	1.5	69 (34)	2.9	2.8	15	291
MC	25 (15)	142 (42)	9	4	26 (10)	1.0	57 (30)	1.6	2.6	7	270

*Eumonhystera filiformis* (Bastian, 1865), *Mononchus truncatus* (Bastian, 1865), and *M*. *sandur* (Eisendle 2008) dominated the MC floodplain sites. *E*. *pseudobulbosa* (Daday, 1896) and *E*. *longicaudatula* (Gerlach and Rieman 1973) were dominant in the habitats of the main channels of the KC, and *E*. *vulgaris* (De Man, 1880), and two *Tobrilidae* species in the KC4 habitat, including *Tobrilus allophysis* (Steiner, 1919), also being highly abundant at KC3. Bdelloid rotifers dominated the rotifer community at all sites. *Mucor* sp., *Fusarium* sp., and *Cladosporium* sp. dominated the fungal community. *Achnanthidium minutissimum* (Kützing) Czarnecki was the dominant species among diatoms. A comprehensive taxon list is provided in Table S3.

### Microbiota

Bacteria were dominated by cocci. Among the flagellates, autotrophic forms prevailed in most of the replicates (93%). The ranking of mean abundances among reaches varied among the microbiota groups (Table 4). Lowest means at the recently emerged MC1 were determined only for bacteria (summed cocci and rods) and UFB. The mean abundances of HNF and ANF (autotrophic nanoflagellates) were lowest at the main channel reaches of the KC and increased toward the MC proglacial (KC1<KC3<KC2<MC1<MC2). Bacteria, UFB, and flagellate (ANF and HNF) abundances were high and followed similar patterns of MC4>MC3>KC4, unlike the abundances of cyanobacteria (MC4>MC1>KC1) and fungi (KC4>MC3>MC2) (Table 4). The seasonal patterns of total abundances also differed between these groups although there was no consistent decrease among and between them at either reach during the summer ablation period GMII (Fig. 3A). The spatiotemporality of the bacteria and nanoflagellate complex was significantly influenced by reach and by the interaction between reach and time, but not by catchment, which had a slightly greater influence than either of the other two factors on the variability in the algae/protist complex (Table 5).

**Table 4:** Mean and standard deviation (±) of abundances of the most important MMB groups, averaged over the sampling period for each reach

	Bacteria	UFB	HNF	ANF	CYAN	CFU	Nematodes	Rotifers	Copepoda	Tardigrades
MC1	1.3 × 10^6^	2.0 × 10^3^	5.7 × 10^2^	4.7 × 10^3^	9.5 × 10^3^	3.69 × 10^1^	3.4 × 10^0^	6.9 × 10^1^	0.0 × 10^0^	6.8 × 10^1^
	(±8.7 × 10^5^)	(±1.9 × 10^3^)	(±7.7 × 10^2^)	(±2.8 × 10^3^)	(±2.3 × 10^4^)	(4.07 × 10^1^)	(±3.3 × 10^0^)	(±5.7 × 10^1^)	(±0.0 × 10^0^)	(±9.3 × 10^1^)
MC2	3.4 × 10^6^	3.2 × 10^3^	9.2 × 10^2^	5.7 × 10^3^	1.3 × 10^3^	4.09 × 10^1^	9.6 × 10^0^	7.3 × 10^1^	3.3 × 10^2^	2.5 × 10^1^
	(±4.0 × 10^6^)	(±4.8 × 10^3^)	(±2.1 × 10^3^)	(±9.3 × 10^3^)	(±3.3 × 10^3^)	(4.42 × 10^1^)	(±6.2 × 10^0^)	(±7.8 × 10^1^)	(±1.3 × 10^1^)	(±4.4 × 10^1^)
MC3	3.4 × 10^7^	9.8 × 10^4^	7.8 × 10^3^	4.0 × 10^4^	1.8 × 10^3^	7.20 × 10^1^	6.1 × 10^2^	1.9 × 10^2^	3.4 × 10^1^	3.9 × 10^2^
	(±3.1 × 10^7^)	(±9.6 × 10^4^)	(±1.0 × 10^4^)	(±3.9 × 10^4^)	(±4.1 × 10^3^)	(1.39 × 10^2^)	(±1.2 × 10^3^)	(±4.7 × 10^2^)	(±1.1 × 10^0^)	(±1.2 × 10^3^)
MC4	6.6 × 10^7^	3.2 × 10^5^	1.1 × 10^4^	8.5 × 10^4^	4.4 × 10^4^	3.28 × 10^1^	1.9 × 10^3^	3.1 × 10^2^	8.1 × 10^1^	6.3 × 10^2^
	(±2.5 × 10^7^)	(±1.8 × 10^5^)	(±1.0 × 10^4^)	(±3.0 × 10^4^)	(±3.2 × 10^4^)	(2.34 × 10^1^)	(±1.2 × 10^3^)	(±2.3 × 10^2^)	(±2.0 × 10^0^)	(±2.4 × 10^2^)
KC1	1.4 × 10^6^	3.3 × 10^3^	1.5 × 10^2^	1.3 × 10^3^	6.5 × 10^3^	2.42 × 10^1^	9.0 × 10^1^	1.3 × 10^2^	1.9 × 10^1^	1.1 × 10^1^
	(±1.7 × 10^6^)	(±4.7 × 10^3^)	(±1.9 × 10^2^)	(±1.4 × 10^3^)	(±1.4 × 10^4^)	(3.30 × 10^1^)	(±2.9 × 10^2^)	(±2.6 × 10^2^)	(±6.9 × 10^1^)	(±3.8 × 10^1^)
KC2	2.2 × 10^6^	7.4 × 10^3^	4.7 × 10^2^	2.1 × 10^3^	2.9 × 10^3^	3.56 × 10^1^	1.4 × 10^2^	1.9 × 10^2^	1.5 × 10^1^	4.4 × 10^0^
	(±2.8 × 10^6^)	(±8.8 × 10^3^)	(±5.2 × 10^2^)	(±2.0 × 10^3^)	(±4.1 × 10^3^)	(4.70 × 10^1^)	(±1.9 × 10^2^)	(±2.1 × 10^2^)	(±3.4 × 10^1^)	(±5.2 × 10^0^)
KC3	1.4 × 10^6^	6.7 × 10^3^	4.4 × 10^2^	1.^6^ x 10^3^	2.6 × 10^3^	1.57 × 10^1^	2.5 × 10^1^	1.4 × 10^2^	1.3 × 10^0^	5.0 × 10^0^
	(±1.7 × 10^6^)	(±6.8 × 10^3^)	(±5.4 × 10^2^)	(±8.9 × 10^2^)	(±9.0 × 10^3^)	(2.05 × 10^1^)	(±2.1 × 10^1^)	(±1.2 × 10^2^)	(±1.6 × 10^0^)	(±8.1 × 10^0^)
KC4	2.3 × 10^7^	6.5 × 10^4^	7.3 × 10^3^	1.8 × 10^4^	4.3 × 10^2^	1.12 × 10^2^	1.1 × 10^3^	2.5 × 10^3^	9.1 × 10^2^	2.5 × 10^2^
	(±2.1 × 10^7^)	(±4.7 × 10^4^)	(±7.9 × 10^3^)	(±9.0 × 10^3^)	(±1.0 × 10^3^)	(7.83 × 10^1^)	(±8.8 × 10^2^)	(±2.4 × 10^3^)	(±1.0 × 10^3^)	(±3.0 × 10^2^)

UFB, unbranched filamentous bacteria; HNF, heterotrophic nanoflagellates; ANF, autotrophic nanoflagellates; CYAN, cyanobacteria; CFU, colony-forming units of fungi. Bacteria, cyanobacteria, and flagellates per cm^2^ surface and mL sediment; invertebrates per 10 cm^2^ and mL sediment; fungi per 10 cm^2^.

**Table 5:** Output of the PERMANOVA for the respective groups: Pseudo-F values are shown for the factors reach (R), date (D), and catchment (CA) and their interactions. Values in italics indicate the contribution of estimated variations derived from the respective factor

	R	R × D	D	CA	CA × D	*CA*	*CA ×* *D*	*R*	*R × D*	*D*	*Res*
Microbiota	27.9**	2.4**	1.7	Pooled	1.2	*–*	*3*	*32*	*12*	*5*	*21*
Fungi CFU	7.0**	3.2**	1.3	1.1	4.5	*2*	*16*	*13*	*14*	*3*	*21*
Algae/protists	2.3**	1.2	2.7**	3.3**	1.2	*26*	*17*	*27*	*15*	*17*	*44*
Invertebrates	33.6**	2.0**	2.3	Pooled	1.4	*–*	*5*	*35*	*11*	*6*	*24*
Nematodes	12.9**	2.1**	1.6	1.7	1.4	*17*	*9*	*39*	*21*	*8*	*42*
Rotifers	11.6**	1.5	2.4	1.5	1.5	*11*	*7*	*30*	*11*	*8*	*30*

Significance in the rejection of H0 at **P ≤ 0.01. Number of permutations = 9999.

### Invertebrates

Rotifers and nematodes dominated the invertebrate fauna. Copepods and macroinvertebrates such as chironomids or planarians (data not shown) were extremely scarce not only at all MC reaches but also along the main channel of the KC (Table 4). Copepods were only present with considerable abundances at KC4. Nematode and rotifer abundances were lowest at the harsh proglacial (MC1<MC2), whereas copepod abundances were lowest at MC1 and KC3 and tardigrades ranked totally differently (KC2<KC3<KC1). The rankings of the highest abundance differed between groups and could not be consistently ascribed to either deglacierization stage or habitat conditions: MC4>KC4>MC3 (nematodes), KC4>MC4>MC3 (rotifers), and KC4>KC1>KC2 (copepods). Spatiotemporal abundances did not undergo a uniform decline in summer (Fig. 3B). Analysis of the complex community structures by resemblance matrices within the PERMANOVA analysis showed that while for nematodes reach and an interaction between reach and date were relevant, only reach explained the significant differences in rotifer resemblance patterns; catchment had no significant influence on either group (Table 5).

### Relation between abiotics and community patterns

The relations between the GI_m_ and the community characters that fulfilled the regression requirements (18) are listed in Table 6. The GI_m_ explained >70% of the variations in nematode maturity and abundances and >50% of the variations in six other characters (e.g., nematode, rotifer, and diatom taxonomic richness). Six factors did not significantly alter in parallel with changes in the GI_m_. WD correlated negatively with tardigrades, HNF (both *P* < 0.01), nematodes, and copepods (both *P* < 0.05); WV negatively with nematodes, tardigrades, bacteria, threads, HNF, ANF (all: *P* < 0.01), rotifers, and cyanobacteria (both *P* < 0.05). A significant positive correlation was established among invertebrates groups and among microbiota morphotypes (*P* < 0.01) (all Spearman's rho). Finally, according to the BIOENV, the variability in BI alone was most strongly related to the complex variability in the MMB resemblance patterns (Spearman's rho: 0.54; *P* = 0.01, number of permuted rho's >global rho = 0).

**Table 6:** Results of the regression analysis (*R*^2^, *F*, *b*0, and *b*1 as the regression coefficients of the linear functions)

	*R*^2^	*F*	*b*0; *b*1
Nematode MI	0.74**	63.78	2.36; 0.29
Nematode TR	0.53**	23.40	17.18; 4.72
Nematode H′	2.E-03^**0**^	0.04	1.88; −0.02
Nematode M_L10	0.77**	71.09	1.94 0.84
Rotifera TR	0.62**	34.00	9.09; 4.71
Rotifera H′	0.67**	41.99	0.59; 0.49
Rotifera M_REZ	0.13^**0**^	3.32	0.01; −0.01
Bacteria M_L10	0.53**	23.23	6.68; 0.54
HNF M_L10	0.42**	14.96	3.01; 0.53
Diatoms TR	0.64**	36.69	30.50; 13.69
Algae TR	0.27*	7.77	6.78; 2.71
ANF M_L10	0.23*	6.41	3.76; 0.41
Cyanobacteria M_L10	8.E-08^**0**^	0.00	2.48; −0.02
UFB M_L10	0.62**	34.25	4.09; 0.69
Algae M_L10	0.29**	8.58	4.20; 0.95
Protists M_L10	0.02^**0**^	0.46	1.63; 0.13
Protists TR M_L10	0.11^**0**^	2.69	0.75; 0.45
Fungal CFU M_L10	0.12^**0**^	2.97	2.19; 0.15

MI, maturity; TR, taxonomic richness; H′, Shannon diversity; M, mean abundance; L10, log_10_-transformed data; REZ, reciprocal value; UFB, unbranched filamentous bacteria.

**Highly significant: *P* < 0.01, *significant: *P* < 0.05 and ^0^no significant relation between the GI_m_ and the respective community parameters.

## Discussion

### General aspects

The distinctly different past and present characteristics and activities of the studied glaciers at the two catchments manifested as distinct habitat templates within a range of extremely rough to benign conditions. These conditions were condensed into three groups by the GI_m_: extremely harsh (MC1 and MC2), harsh (KC1–KC3), and benign (MC3, MC4, and KC4). The MC is representative of a high degree of glacierization, but also of a glacier (Pasterze glacier) that has rapidly decreased in size during the last few decades. The latter accounts for the two distinct sections of the MC: (1) a highly unstable proglacial, comprising the glacier source (MC1) and MC2, which consists mainly of unconsolidated moraine material, and (2) the consecutive silted floodplain area (Krainer and Poscher 1992). The ongoing rapid loss of ice masses during the study period (Fischer 2010) led to a strong glacier influence at the proglacial (e.g., low water temperature and a low GI_m_) that was distinctly modified along the silted floodplain area (e.g., high water temperature and high GI_m_). Such silted floodplain areas can often be ascribed to large melt water streams (Krigstom 1962; Hodgkins et al. 2009).

In contrast, the KC is a typical example of a less glacierized catchment where the accumulation area now predominates after the wide and effective retreat of the ablation area. It is also representative of the fragmentation of a once contiguous glacial surface, as is similarly expected for many glaciers in response to predicted climate changes (Jiskoot and Müller 2012). This fragmentation, resulting partly in cliff glacier remnants, was responsible for habitat conditions that contradicted a longitudinal gradient of amelioration along the KC main channel sites, particularly during the melting period. Cliff remnants at KC3 intruded upon a proposed amelioration gradient, as their summer-time effluents caused high current-water velocities at KC3. These findings suggest that the downstream development of glacier streams as a consequence of ongoing glacier retreat will abrogate any downstream longitudinal increase in benignity in glacier rivers (see Milner et al. 2001) when a stream accompanying the glacier remnants develops in parallel.

One of the basic assumptions in this study, namely, that the two catchments cause significant differences in the MMB because of differences in their harshness – defined according to the percentage of glacierization, glacier retreat patterns, and stream ages – was not confirmed. Nevertheless, single community descriptors (e.g., algal, protist, nematode, and rotifer species numbers; rotifer and nematode H′ diversity; and nematode maturity), might better capture the harshness of the catchments, as evidenced by the lower values in the MC, indicative of higher overall catchment harshness. Structural alterations in the macrozoobenthos were shown to occur with decreasing glacerization, with an increase in the numbers of individuals and species with ongoing glacier retreat in alpine areas (Füreder 2007). Our study comparing the KC and MC is the first to analyse similar causalities for species number of the micro- and meiozoobenthos, comprising protists, rotifers, and nematodes. Thus, as for the macrofauna, we also found evidence of an increase in the taxonomic richness with decreasing glacierization, at least to the extent occurring between the two catchments (from 60% to 25%).

Between-catchment comparisons, as discussed above, are generally rare in glacier river studies. More often, the focus is on the distribution of community patterns (abundances, richness) as a function of varying habitat harshness along three main axes: (1) the longitudinal decrease in harshness with increasing distance from the glacier margin, which should typically be paralleled by an increase in stream age; (2) a seasonal decrease in harshness outside the glacier ablation period (1 and 2: Ward 1994; Milner et al. 2001); and (3) a lateral component enabling the existence of less extreme rhithral, glacio-rhithral, and krenal habitats (Tockner and Malard 2003; Uehlinger et al. 2003). Most of the studies referring to these axes have reported an increase in structural complexity with respect to the diversity and/or abundances of their studied organisms together with a decreases in harshness (Battin et al. 2001; Lods-Crozet et al. 2001a,b; Robinson et al. 2001; Burgherr et al. 2003; Bürgi et al. 2003; Malard et al. 2006; Eisendle 2008). However, observations refuting these basic schemes have also been made, for example, a report of a decrease in fungal species numbers in the river downstream (Gessner and Robinson 2003) and the results of this study (but see below).

In this study, taxon numbers of benthic fungi and of most of the other groups decreased (see Table 3) downstream from the MC glacier source to MC2, and from KC2 to KC3 although by different amounts. MMB abundances also partly decreased in the river downstream along the MC and KC main channel sites. These observations deviate from the abovementioned scheme in glacier river research, in which a longitudinal downstream increase in taxonomic richness and abundances in relation to downstream habitat amelioration and succession progress (stream age). However, these deviations are consistent with the particular catchment characters in this study, as already discussed herein, namely, the instability of MC2 and the lengthened, glacier-accompanied main channel course of the KC. Finally, to answer the question whether harshness or stream age has greater determining relevance for the MMB, comparisons among KC3 and KC4, as well as KC1 and MC2, and KC4 and the sandur complex lead to the conclusion that benignity and harshness are more important than stream age, both within and between catchments.

In its original form, the GI was introduced to improve the relational analysis of glacier influence on macrozoobenthic patterns (Ilg and Castella 2006). The GI integrates within-stream conditions that change with varying glacier influence (water temperature, EC, BI, and turbidity). Despite several shortcomings, the index is relatively easy to apply and, in most cases it distinguishes complex habitat conditions, relating them, more or less strongly, to community patterns (Ilg and Castella 2006; Brown et al. 2010). The reasonable reach grouping confirmed the utility of the modified version developed for this study. More importantly, perhaps, ours is the first application of the GI to the MMB and it revealed well-established relationships for most of the micro- and meiobiota characters. These relationships were often stronger than those previously reported for the few macroinvertebrate structural parameters (richness, abundances) examined thus far (Brown et al. 2010). Specifically, the GI_m_ clearly indicated the positive response of certain community parameters (e.g., taxonomic richness of nematodes, diatoms, and other algae; abundances of bacteria, flagellates, algae, and nematodes) to decreasing glacier influence, which in itself entails complex and reach specifically altered conditions between and among catchments.

Predictions on the nature of the MMB changes along glacier-dependent river systems, however, should be generalized only with caution, based on the varying results described herein for the different groups. A summary of the results obtained for relation patterns between the MMB and several abiotic parameters clearly showed, for example, that an increase in water temperature is insufficient to promote an increase in the abundance or taxonomic richness of most groups when it is accompanied by high-current velocity and low-bed instability. The predicted summer-time shift to precipitation-driven effluents in alpine catchments as discussed in Milner et al. (2009) might therefore have similar repressive effects on MMB richness and density, due to their flashy, erosive, and less predictable run-off character. A shift of water sources might also bear serious consequences for catchment functionality if there is a loss of benign habitats in response to a loss of groundwater recharge function during the ablation period (Tockner and Malard 2003). It is particularly these habitats that, regardless of their bed sediment texture, are important local hot spots of diversity and thus of functionality.

### Selected community aspects

This study adds new and important information regarding the autotrophic component of glacier-fed rivers. Our results suggest that high-diatom species richness is not restricted to the epilithon or to those habitats with diminished glacial influence and are thus in contrast to previous studies (see Rott et al. 2006). In particular, the number of diatom species at silted floodplains highlights the need for greater attention to these habitats in future research. However, it is not only diatom species numbers that seem to be distinctly underrepresented when exclusively determined on epilithic substrates, as indicated by other algal species number among KC reaches (compare Bürgi et al. 2003). A comparison of the photoautotrophic components among the MC and KC reaches showed the delayed succession of a diverse autotrophic community not limited to diatoms in riverine habitats shaped by rapid deglacierization. Finally, ANFs, although widely neglected, may be important primary producers in glacier-fed river habitats. As a subject of cryoconite research, their general ecological importance within glacier biomes has already been demonstrated (Hodson et al. 2008; Anesio et al. 2009). Based on their distribution patterns, as determined in our study, ANFs can be considered as a constant and seemingly adaptive component within carbon cycling processes in glacier-fed river habitats driven by varying glacier influences.

Despite consistently strong differences in the thermal and hydrological regimes of the two catchments, there were no significant corresponding differences in the resemblance patterns of the bacterial and flagellate microbiota. Specifically, microbiota densities might be positively rather than negatively affected by a huge loss of ice masses. This conclusion is based on the abovementioned growing appreciation of glaciers as important habitats of microbial organisms, thereby fulfilling important ecological functions on a regional as well as a global scale (Kastovska et al. 2007; Mindl et al. 2007; Hodson et al. 2008; Anesio et al. 2009; Stibal et al. 2012; Viles 2012). Bacteria have been shown to rapidly alter their genetic expression patterns, changing from swimming to sessile life forms (Hall-Stoodley et al. 2004). This could be advantageous in shifts from glacial to riverine benthic habitats, such as by facilitating colonization at recently deglacierized sites, in favoring downstream drift, and in the recolonization of unstable habitats.

The distribution patterns of the fungal CFU counts and fungal species numbers did not show a uniformly increasing trend in the shift from harsh to benign conditions or from high to low glacierization, suggesting their independence from varying glacial influence and thus from glacially driven hydrological and thermal regimes. The taxa from the benthic substrate clearly differed from those described in glacier river studies on fungi, in which leaf-litter traps were used (Gessner and Robinson 2003). The MC and KC taxa partly resembled the spectra of terrestrial taxa related to understory vegetation (De Bellis et al. 2007), corroborating the assumed role of fungi in connecting aquatic habitats and their catchments (Jobard et al. 2010) but also implying changes in the benthic fungal community in response to those in the surrounding vegetation (Walther et al. 2005; Parmesan 2006; Leonelli et al. 2011). The observed fungal patterns might reflect the importance of fungi as an essential nutrient resource in high-altitude reaches, particularly for smaller invertebrates (e.g., nematodes and rotifers), by providing suitable high-energy and high-quality “mouthfuls” of food. This, together with the reliance of the fungal taxa on their surroundings, would add a further, previously unrecognized component to both the intrinsic relationships between potential resources and their consumers and the alterations thereof by ongoing changing environmental conditions.

Among those invertebrates for which species composition was investigated, habitat conditions and deglacierization stages contributed less to the similarity patterns of rotifers than of nematodes. This finding can be explained by the clear predominance of bdelloid rotifers, a group particularly well adapted to hardy conditions and with a number of pioneer species (Wallace et al. 2006; Hodson et al. 2008; Webster-Brown et al. 2010). Both the behavioral adaptations of monogononts searching for shelter in deeper interstitial layers (Schmid-Araya 2000) and the diurnal migrations of monogononts and nematodes aimed at high-flow avoidance (Smith and Brown 2006) would account for the maintenance of low, but permanent densities of these groups despite frequent high-flow conditions. These activities would also provide a behavioral advantage for these organisms in the future in response to climate change, when they are potentially confronted with an increased glacial melt and the prolongation thereof as well as less predictable precipitation flood events.

Although less abundant at particularly harsh sites, nematodes were more diverse than rotifers at each reach and catchment, with the highest abundances at the extremely differently structured benign sites. Therefore, nematodes might be able to well cope with a variety of reach conditions, a conclusion supported by the variety of running waters harboring nematodes (Eisendle 2009 and references therein). The nematode resemblance patterns clearly differentiated between the different KC and MC habitat types, and the nematode maturity was related to the GI_m_-established reach grouping. Furthermore, the observed nematode feeding types (data not shown; Yeates et al. 1993), were in good accordance with the respective food resources of each investigated reach. Together, these results support the usefulness of nematodes as indicators of glacially induced habitat templates as well as the development of benthic resource variety along glacier-fed rivers. Finally, we tentatively predict the loss of *Hofmaenneria hazenensis* (Mulvey 1969) (first recorded for the Arctic), with the loss of glacial influence, because in this study it inhabited only those river sites with distinct surface glacier influences (all except KC4).

## Supporting Information

**Table S1.** Methods of preservation and laboratory treatment.

**Table S2.** Taxon list.

**Table S3.** PERMANOVA (pairwise) test results of seasonal differences in the MMB groups.
